# Human genetics and genomics research in Ecuador: historical survey, current state, and future directions

**DOI:** 10.1186/s40246-019-0249-8

**Published:** 2019-12-10

**Authors:** Marlon S. Zambrano-Mila, Spiros N. Agathos, Juergen K. V. Reichardt

**Affiliations:** 1 School of Biological Sciences and Engineering, Yachay Tech University, San Miguel de Urcuquí, Ecuador; 2 Bioengineering Lab, Earth & Life Institute, Université Catholique de Louvain, Louvain-la-Neuve, Belgium; 3 Australian Institute of Tropical Health and Medicine (AITHM), James Cook University, Smithfield, QLD Australia

**Keywords:** Human genetics, Human genomics, South America, Ecuador, PubMed, Scopus, Google Scholar, Web of Science, Database search

## Abstract

**Background:**

In South America, the history of human genetics is extensive and its beginnings go back to the onset of the twentieth century. In Ecuador, the historical record of human genetics and genomics research is limited. In this context, our work analyzes the current status and historical panorama of these fields, based on bibliographic searches in Scopus, Google Scholar, PubMed, and Web of Science.

**Results:**

Our results determined that the oldest paper in human genetics coauthored by an Ecuadorian institution originates from the Central University of Ecuador in 1978. From a historical standpoint, the number of articles has increased since the 1990s. This growth has intensified and it is reflected in 137 manuscripts recorded from 2010 to 2019. Areas such as human population genetics, phylogeography, and forensic sciences are the core of genetics and genomics-associated research in Ecuador. Important advances have been made in the understanding of the bases of cancer, some genetic diseases, and congenital disorders. Fields such as pharmacogenetics and pharmacogenomics have begun to be explored during the last years.

**Conclusions:**

This work paints a comprehensive picture and provides additional insights into the future panorama of human genetic and genomic research in Ecuador as an example of an emerging, resource-limited country with interesting phylogeographic characteristics and public health implications.

## Background

In recent years, the understanding of the genetic bases of complex diseases such as heart diseases, diabetes, and cerebrovascular disorders has notably grown thanks to human genetics and human genomics research worldwide [1–6]. Whereas the history of human genetics investigations in Latin America is broad, the historical record of human genomics research is more limited and recent. The beginning of medical genetics in the region was marked by Luis Morquio, who worked on mucopolysaccharidosis in 1929, in Uruguay [7]. From the Ecuadorian standpoint in human and medical genetics, three facts are important to mention: first, the oldest scientific document about human genetics in Ecuador was the book “La genética y el hombre” (*Genetics and Man*) published by Robert Hoffstetter in 1947 [8]. Second, the Ecuadorian Society of Human Genetics (SEGH) was legally constituted in 1994 [9]. Third, the Genomics and Genetics Network [“Red de Genética y Genómica” (ReGG)] in Ecuador was legally established in 2018.

Recent efforts, such as the book “Genética en el Ecuador: 30 años” (*Genetics in Ecuador: 30 years*), published in 2016, has collected historical data of scientific manuscripts related to medical genetics in Ecuador [9]. In this document, the authors identified a total of 354 books and 259 scientific articles of medical genetics–associated works from 1984 to 2016 present in different databases [9]. Even though this work gives us an overall insight into the history and current state of medical genetics, many gaps still remain in human genetics and genomics research in Ecuador. Data such as the beginnings and the trends of research in human genetics and genomics in Ecuador, the distribution of human genetic and genomic research across different associated medical areas, plus the quality and impact of the Ecuadorian scientific articles published in different peer-reviewed journals are still unknown and this phenomenon can be attributed to the limited access to the historical record and the lack of local databases in Ecuador.

To examine the historical context and current landscape of human genetics and human genomics in Ecuador, we performed a detailed analysis of research in these areas based on bibliographic searches in PubMed, Scopus, Google Scholar, and Web of Science. In this context, we scored scientific articles published in peer-reviewed journals and co-authored by investigators from Ecuadorian institutions. The aim of this paper is to establish the current status and historical research trends of human genetics and human genomics in Ecuador. Furthermore, our study attempts to analyze research across different health and medical areas, and human diseases so it may enable to precisely determine how the research in human genetics and human genomics is distributed and concentrated within the health care context of Ecuador as an example of an emerging, resource-limited country.

## Results

### Relevant articles identified in Scopus, PubMed, Google Scholar, and Web of Science

The keywords “Human Genetic Ecuador” and “Human Genomic Ecuador” in all search engines produced initially 40,506 hits. After the scrutinization process, the primary bibliographic searches for both “Human genetic Ecuador” and “Human genomic Ecuador” in Google Scholar, PubMed, Scopus, and Web of Science provided 38,890; 553; 386; and 677 results, respectively (Table 1). These data sets were scrutinized based on two principles: first, their relationship to human genetics and genomics and second, articles published by authors belonging to Ecuadorian institutions. In this context, PubMed provided a total number of 133 articles of interest, which were retrieved from 553 records identified in the primary search (Table 1), whereas in Web of Science, 90 manuscripts were recovered from 677 records. Additionally, the search in Scopus resulted in 120 publications of interest retrieved from 386 publications whereas in Google Scholar, the research produced 275 relevant records obtained from 38,890 publications in the primary search. Based on its search efficiency of 31.08%, Scopus was the most useful search engine in terms of yield to seek out publications in both fields, human genetics, and human genomics, in Ecuador (Table 1). In contrast, Google Scholar with a search efficiency of 0.71% was the least effective in terms of pertinent results per analyzed data quantity which could be attributed to the search methodology (search per year). It is worth mentioning that there were several common articles for at least two databases found as either “Human Genetic Ecuador” or “Human Genomic Ecuador,” depending on the search engine. This trend may be attributed to the overlap of field of study within these biomedical areas so that it was necessary to build up a record of unique articles for subsequent analysis.

**Table 1:** Number of articles analyzed and number of relevant manuscripts recorded in PubMed, Scopus, Google Scholar and Web of Science

Database	Analysis and processing of data	Type of search		Total number of articles	Search efficiency* (%)
		“Human genetic Ecuador”	“Human genomic Ecuador”		
PubMed	Articles analyzed	295	258	553	24.05
	Relevant articles in human genetics/human genomics	52	81	133	
Scopus	Articles analyzed	349	37	386	31.08
	Relevant articles in human genetics/human genomics	103	17	120	
Web of Science	Articles analyzed	643	34	677	13.30
	Relevant articles in human genetics/human genomics	81	9	90	
Google Scholar	Articles analyzed	27,557	11,333	38,890	0.71
	Relevant articles in human genetics/human genomics	157	118	275	

*Search efficiency of each database is the ratio between number of interest articles and number of analyzed articles. Subsequently, these proportions were transformed to percentage in order to standardize the data

### Survey of human genetics and human genomics in Ecuador

**Fig. 1:**
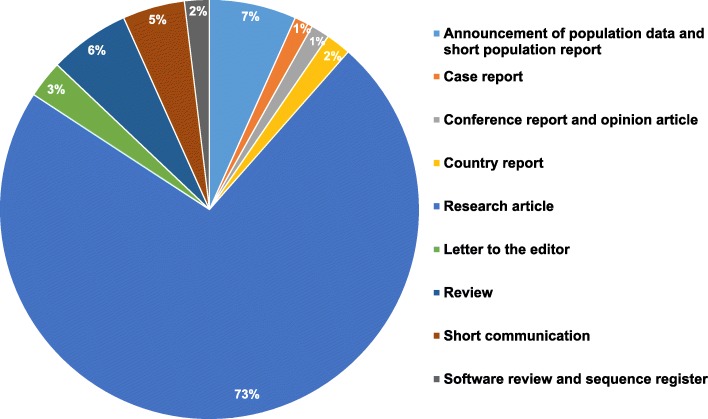
Ecuadorian scientific output associated with human genetics and human genomics classified according to the types of scientific articles

Our final definitive record, which consisted of 209 unique articles of interest retrieved from the three different search engines, is summarized in Fig. 1. Our bibliographic register includes 152 primary research articles, 14 announcements of population data and short population reports, 13 reviews, 10 short communications, 6 letters to the editor, 4 country reports, 4 software reviews and sequence registers, 3 case reports, 2 conference reports, and 1 opinion article.

**Fig. 2:**
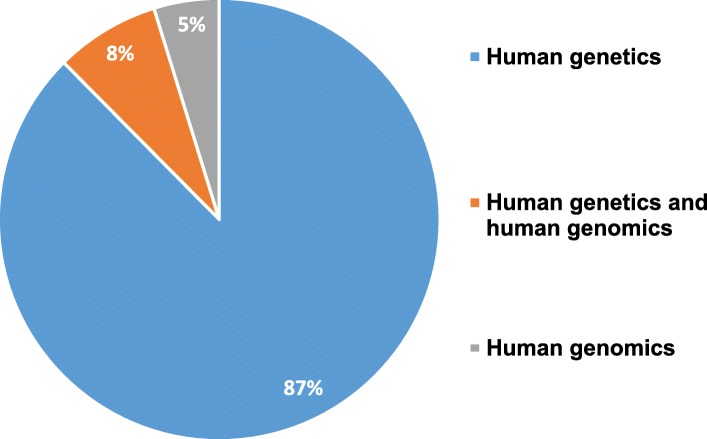
Percentage of articles, which were retrieved from Scopus, PubMed, Google Scholar, and Web of Science, classified as human genetics, human genomics, or both in Ecuador

Subsequently, these manuscripts were reclassified according to their potential direct relationship to human genetics, human genomics, or both in order to normalize all data. Our research shows (Fig. 2) that 183 articles representing 87% of all publications fall into human genetics research. In contrast, only 16 articles, 8% of the whole set, include human genetics research using genomic techniques such as whole genome sequencing, exome sequencing, transcriptome sequencing, microarrays, etc. Finally, only 10 articles, or 5 % of the entire record, are directly related to human genomics research in Ecuador. This situation reflects that the current research technologies, especially those associated to human genomics, have been largely underutilized in an emerging country like Ecuador. This latter phenomenon has been attributed to the limited funding, the elevated cost of setting up and supporting a research facility, the absence of experienced personnel, the restricted availability of tools for data use and analysis, the lack of a well-established regulatory frame, and even a restricted access to updated proper literature (Helmy, Awad, & Mosa, 2016). However, this Ecuadorian reality opens new future lines of research not only in this relatively unexplored area in the country but also in associated biomedical and other fields.

### Historical outlook of human genetics and human genomics research

In Ecuador, the beginning of research in human genetics and genomics is relatively recent compared to other countries in South America [7]. In the region, the onset of clinical genetics can be traced back to 1929 with Luis Morquio, who worked on mucopolysaccharidosis in Uruguay [7, 10]. In Argentina, Sáez and Etcheverry worked on cytogenetics in 1934 and on the rhesus blood group system in 1945, respectively [7]. In Ecuador, Leone and Paz-y-Miño (2016) showed that the oldest scientific article is “Introducción a la ecología médica ecuatoriana I parte” (“Introduction to Ecuadorian medical ecology–part I”), published in the Ecuadorian Cultural Center’s bulletin (Boletín Científico Casa de la Cultura Ecuatoriana) in 1984 [9]. However, our own survey determined that the oldest scientific article found in Scopus and Google Scholar was “The multinational Andean genetic and health program: II. Disease and disability among the Aymara” published in 1978 and whose authors include Biffret Díaz, Daniel Gallegos (affiliated to the Central University of Ecuador), Federico Murillo, Edmundo Covarrubias, Thusnelda Covarrubias, Roberto Rona, William Weidman, Francisco Rothhammer, and William J. Schull [11].

**Fig. 3:**
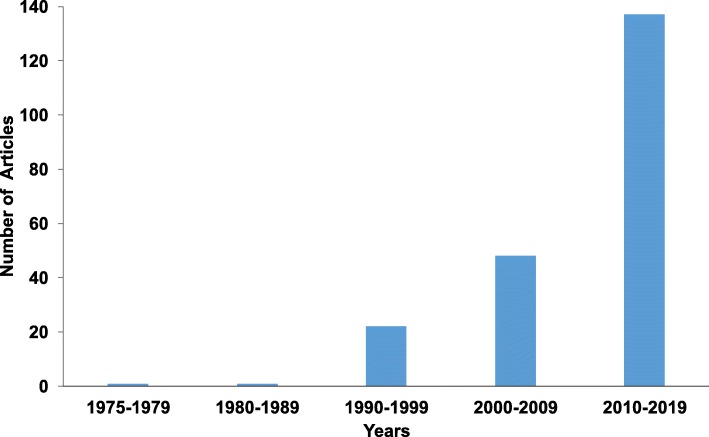
Trendline of Ecuadorian scientific production in human genetics and human genomics since 1978 based on data retrieved from PubMed, Scopus, Google Scholar, and Web of Science

Recent studies have shown that scientific publications in conventional peer-reviewed journals have significantly increased worldwide [12]. Countries with an expanding economy have ramped up their production of science, innovation, and technology [13]. Figure 3 reports that the number of publications in both human genetics and human genomics has increased during the last couple of decades in Ecuador. From a historical standpoint, our survey determined 24 articles published from 1978 to 1999, 48 articles published from 2000 to 2009, and the number of publications from 2010 through June 2019 is 137. In this context, the growth rate of human genetic research is more noticeable after the 1990s because previous numbers are relatively inconsequential. This latter situation may be attributed to the fact that the databases analyzed do not include certain Latin American journals and Ecuadorian journals. Since 2010, there has been a pronounced increase in the number of scientific publications of both fields in conventional peer-reviewed journals compared to previous decades. SCImago Journal and Country Rank (SJR), which is an evaluation portal for journals and countries based on data contained in the Scopus database since 1996, support our inferences about Ecuador as a promising science generator by placing Ecuador in 87th place among 239 countries, in regard to research in clinical genetics and medicine, in 2016 (SCImago Journal & Country Rank, 2019a). Additionally, this is consistent with a very recent analysis which found that research productivity in Ecuador increased by more than five-fold from 2006 to 2015 across the ten most represented scientific fields in the country [13].

**Fig. 4:**
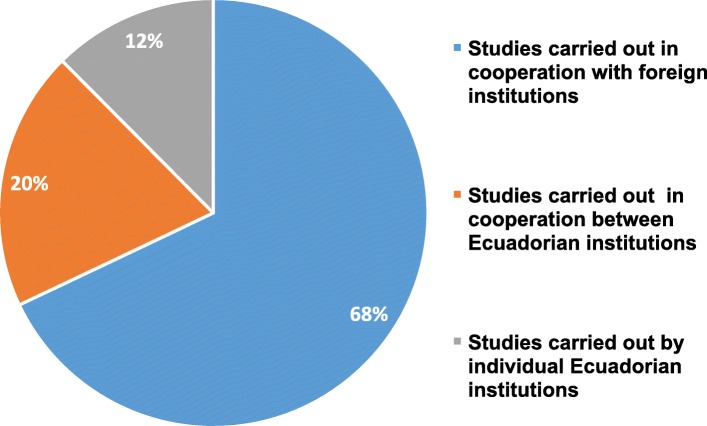
Classification of Ecuadorian scientific output associated to human genetics and genomics based on international or national co-authorship of articles published in peer-reviewed journals

International cooperation in research has played an important role in Ecuadorian scientific output associated to human genetics and human genomics. The emerging phenomenon of global cooperation in research with industrialized countries has been manifested through large increases in co-publication between Europe and other regions [14]. In Ecuador, this cooperation in science can largely be perceived as a national, regional, and global phenomenon. Figure 4 shows that most of Ecuadorian scientific output (68% of the whole record) associated with human genetics and human genomics has been achieved through joint work involving both Ecuadorian and foreign institutions. Furthermore, Ecuadorian scientific output developed thanks to cooperation among Ecuadorian institutions such as public and private universities and hospitals which represents 20% of the whole record, while 12% of the studies and publications retrieved from the literature has been carried out solely by individual Ecuadorian institutions with great efforts. Global collaboration is growing as a part of all scientific cooperation, as manifested by co-authorships of articles published in peer-reviewed journals worldwide [15]. From the Ecuadorian standpoint, such partnerships allow the increasing costs of research infrastructure and technological facilities to be shared. In addition, this cooperation must be seen as a link which enables Ecuadorian researchers to establish new collaborative projects between different scientific fields. Partnerships in research allow researchers and scholars to become familiar with other research systems and funding opportunities. Finally, this cooperative approach is becoming a tool to draw attention to young and promising Ecuadorian scientists. These realizations are reflected in the recently reported finding that over 80% of Ecuadorian publications in the last decade involve international collaboration [16]. In this connection, it is instructive to compare this trend with the current state of genomic medicine research in neighboring Colombia, where international collaboration is helping local professionals to develop technical, medical, and scientific expertise based on standards of developed countries [17].

### Scientific fields associated to human genetics and human genomics research

**Fig. 5:**
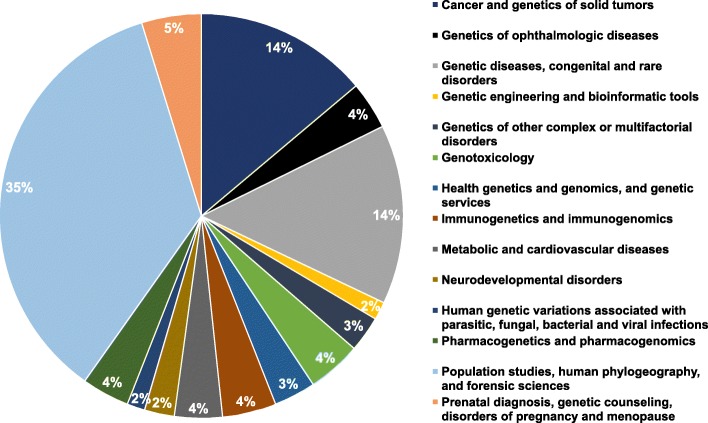
Percentage of articles in human genetics and human genomics associated with different health and medical specialties and human diseases in Ecuador

In Ecuador, human genetics and genomics research has focused on different health and medical areas and many human diseases. Figure 5 summarizes the classification of articles according to the corresponding medical, scientific, or technological area. In spite of the wide range of associated research fields, our study identified the following three dominant scientific and medical areas in which human genetics and genomics research has intensified over the last decades:

1. Population studies, human phylogeography, and forensic science.
2. Genetic diseases and congenital disorders.
3. Cancer and solid tumors.

First, our survey determined that 74 articles, representing 35% of our whole Ecuadorian dataset, are closely related to human population genetics, as well as phylogeography, which describe the human historical processes such as population expansion and migration that influence spatial distributions of contemporary human communities (Fig. 5). Due to overlapping research approaches, this category also covers forensic genetics, including the study of genetic markers which are specific to ethnic subgroups of the Ecuadorian population and can be used for forensic purposes, such as genetic typing of samples from criminal cases, the research of familial relatedness, and the identification of missing persons or victims of disasters [18].

Second, 14% of the whole bibliographic dataset, which represents 30 articles, refers directly to genetic diseases and congenital and rare disorders (Fig. 5). According to the World Health Organization (WHO), congenital disorders annually cause around 303,000 deaths of newborns within the first 4 weeks of birth in the worldwide [19].

Third, 29 publications related to genetics of cancer represent 14% of all documents retrieved from the three databases (Fig. 5). The number of deaths attributed to cancer in the world increased to 8.9 million in 2016 [20]. According to the WHO, the number of new cancer cases is assumed to increase by around 70% over the next 20 years [21]. In this context, all Ecuadorian publications have contributed notable efforts in understanding the molecular bases of cancer over the last few decades [22–25].

It is worth mentioning that relatively novel genetic and genomic research areas are being explored in Ecuador. Our study identified eight articles, i.e., 4% of the whole register, which are directly related to pharmacogenetics and pharmacogenomics (Fig. 5). In Latin America, the development of these fields faces many challenges such as the lack of available clinical laboratories and trained personnel, the low investment in research, and ethical aspects [17, 26]. In spite of these adverse circumstances, these areas have shown expanding trends in health and medical sciences so both efforts and resources should be committed to the development of these fields to guarantee the success of drug development and therapeutics for the Ecuadorian population [27, 28].

## Discussion

### Current research status of human genetics and human genomics in Ecuador

In Latin America, a meaningful increase in both research spending and scientific output has been driven by the dynamic and emerging economies in the region. Brazil dominates as a major player in regional science, whereas Chile shows great advances in patents [29]. Ecuador in terms of medical research including clinical genetics was placed twelfth among 36 countries and dependent territories in Latin America, based on the number of articles published from 1996 through 2018 [30]. Furthermore, both productivity and impact of today’s most active research areas, as cataloged in Scopus, are on the ascendance in Ecuador over the last decade [13, 16]. All of this reflects a promising future of Ecuador as an emerging producer of science in the region [13, 16, 27].

Although the research output in human genetics and human genomics in the region has significantly increased during the last decades, a number of problems still remain. In South America, research impact and quality have not kept pace with growing scientific production so that the region’s research articles still struggle to capture citations from the rest of the world [31, 32]. Second, based on the number of articles published from 1996 through 2018, Ecuador continues to occupy one of the last places with respect to medical research including clinical genetics in 2018, more specifically 8th place among 10 countries in South America [30]. Therefore, it is necessary to develop educational and funding strategies at all organizational levels in order to promote high-quality research in the country, including in-human genetics and genomics along with cognate medical areas.

### Future perspectives

From the genetic standpoint, the ethnic background, as well as ancestry, may have relevance for the understanding of epidemiology and treatment of many disorders affecting Ecuadorian populations [33]. According to the last national census of 2010, the size of the Ecuadorian population was 14,483,499 inhabitants of whom 71.9% self-identified as Mestizos (people of mixed European and Amerindian ancestry); 7.4% as Montubios (half-caste population, of indigenous origin, and inhabiting certain zones in the Ecuadorian coast region); 7.2% as Afro-descendants (African ancestry); 7% as Amerindians; and 6.1% as Caucasians (mostly European ancestry) [34]. Gonzales and López-Pulles (2010) segregated the Ecuadorian population in three major ethnic groups: Mestizos, Amerindians, and Afro-Americans. This ethnic diversity, which has emerged due to ethnically intermixing processes, such as different waves of migration and colonization in history, has built up a particular genetic structure of the Ecuadorian population across the country’s whole territory [35]. As far as ensuring participant safety and research quality, this ethnic complexity in the population makes Ecuador a key country to carry out clinical studies in which the incidence, as well as the prevalence of particular diseases, differs among ethnic subgroups.

Despite the high similarity between individuals’ genetic material, several variants in DNA sequences such as nucleotide polymorphisms, deletions, insertions, and tandem repeats exist across different human populations [36]. Even though there are extrinsic elements such as several demographic and environmental factors which can influence drug response variability, these differences can also be explained through intrinsic aspects such as ethnicity, ancestry, and differences in the expression of drug-metabolizing enzymes or drug transporters, as well as in the expression of drug targets, due to the large genetic variability within a particular population [37]. In this context, novel fields such as pharmacogenetics and pharmacogenomics have opened innovative therapeutic methodologies through the use of current genomics advances such as next-generation sequencing, whole-exome and whole-genome-association studies, preferably including diverse and under-represented individuals, which have led to the understanding of the genomic basis and the genetic etiologies of complex diseases [38]. This latter approach based on identifying pharmacogenetic biomarkers together with the study of pharmacogenetic allele frequencies in different ethnic groups will allow to personalize drug prescription, drug dosing, prevent adverse side effects, and avoid unsuccessful therapies or over-prescription, plus leading to understand the impact of variation in the risk for drug-specific dependence and approaching the molecular and gene-expression mechanisms involved [39]. In this context, precision medicine defined as patient-targeting treatments encourages the use of novel diagnostic and therapeutic methodologies that are personalized to satisfy the patient’s individual needs based on phenotype, pharmacogenetic markers, and even psychosocial aspects [40].

In emerging countries such as Ecuador, the development of fields such as pharmacogenetics and pharmacogenomics has been restricted by scarce research funding, limited knowledge about population gene structure, lack of accredited cell line collections, the lack of highly specialized scientific and technological public or private institutions, the lack of research and working networks in human genomic medicine, and persistent uncertainty about economic and health care benefits, i.e., issues affecting most resource-limited countries [41]. The future outlook for the Ecuadorian health care system should envisage genotype-based therapies based on cost efficiency thanks to rational treatment and the improvement of patients’ quality of life. In this context, future research expectations in human genetics and human genomics should be focused on pharmacogenetics, pharmacogenomics and associated fields, and enabling technologies such as cell sorting, proteomics, metabolomics, and epigenetics in order to promote personalized medicine in diagnosis, drug development, and therapy. Their implementation is a complex political, social, and ethical process in which all stakeholders such as health care institutions, universities, public and private research centers, health care providers, and decision-makers are responsible for carrying Ecuadorian medicine into the genomic era in spite of the scarce resources, in accordance with a “mid-stream” or “fast-second winner” model of innovation [27]. This hopeful model allows a developing country to learn from other countries’ mistakes upstream in basic research and work its way from multiple entry points based on the local characteristics towards translational and clinical implementation commensurate to the country’s limited resources [27, 41]

## Conclusions

Research in human genetics and genomics in Ecuador emerged relatively late compared to other South American countries in the 20th century. In Ecuador, the oldest documented record in human genetics can be dated back as far as 1947, whereas the oldest article published in a peer-reviewed journal is traced back to 1978. A significant rise in human genetics research output can be noticed since the 1990s and its growth rate has substantially increased since 2007. In contrast, recent efforts in human genomics have resulted in limited numbers of publications included in the databases analyzed.

Scientific areas such as human population genetics, phylogeography, and forensic sciences are hot topics of gene and genome-related studies during the last decades. Furthermore, significant advances have been made in terms of understanding the molecular and genetic bases of some types of cancer, some genetic diseases, and congenital disorders. Additionally, novel fields such as pharmacogenetics and pharmacogenomics have started being explored lately.

In spite of this promising reality, the quality of the publications and the impact of the research are critical so they must be evaluated in depth and strengthened in Ecuador. In this context, the current survey of human genetics and genomics research in Ecuador is encouraging and indicates that the Ecuadorian research in the mentioned fields is poised to expand further to contribute to the country’s needs as well as Latin America’s and perhaps the world’s knowledge base.

A plurality of Ecuadorian stakeholders including, most notably, academic researchers and clinical practitioners have charted the course of the research in human genetics and human genomics. The current necessities and gaps can be filled through strengthening national and international collaborations and partnerships in which the cost of the infrastructure and technological facilities can be shared and the researchers and institutions can look for joint and novel projects. The promotion of collaboration networks is a great opportunity not only for the Ecuadorian research establishment to get to know other research systems and funding opportunities but also to recruit young and promising Ecuadorian scientists.

## Methods

### Retrieving articles from PubMed, Google Scholar, Scopus, and Web of Science

Our literature survey included primary research articles, announcements of population data, short population reports, reviews, short communications, letters to the editor, country reports, software reviews, case reports, conference reports, opinion articles, and sequence registers in PubMed, Google Scholar, Scopus, and Web of Science through June 2019. The keywords were “Human Genetic Ecuador” and “Human Genomic Ecuador” and the searches were done via adjusting “custom range” to limit the searches per year and “sort by relevance” that was the criterion employed for “sorting order.”

### Processing and categorizing articles

1. Article types published in peer-reviewed journals.

The publications in the final record were categorized depending on article type based on the criteria of each peer-reviewed journal. The following classes were considered:

- Research article including novel and original primary research works.
- Announcement of population data and short population report, articles aimed at the publication of population data of human polymorphisms, and manuscripts focusing on immunogenomic population data.
- Review summarizing insights or findings in fields associated to human genetics and genomics.
- Short communication, short papers addressing controversial ideas and novel findings and do not meet the “research article standards.”
- Letter to the editor focusing on analysis of previously published articles or short articles of broad interest for the journal’s readers.
- Country report, short announcements of research results related to status and the historical perspective of a medical or scientific field in a country.
- Software review and sequence register, reports about novel bioinformatic tools or available software and reports of nucleotide sequence data.
- Case report, well-documented medical and clinical reports of cases including disease, symptoms, signs, diagnosis, and treatment in individual patients.
- Conference report and opinion article not only show key findings and conclusions presented and discussed at conferences but also include opinion manuscripts about the current high-impact findings in the relevant scientific area.

2. The direct relationship between publications and human genetics, human genomics, or both fields

Subsequently, by analyzing the title, abstract, and its keywords, each article was classified according to its research relationship with human genetics, human genomics, or both fields. The term “relationship” puts emphasis on the research approach or aim, the research methodology and technology (such as whole genome sequencing, whole exome sequencing, etc.) used to carry out the experimental study.

3. Research encompassing interinstitutional cooperation

To understand the role that the research cooperation between Ecuadorian institutions and foreign institutions has played in the development and growth of human genetic and genomic research, the articles were classified in accordance with the affiliations of all authors.

- “Studies carried out in cooperation between Ecuadorian institutions” involves publications whose authors’ affiliations showed more than one Ecuadorian institution.
- “Studies carried out in cooperation with foreign institutions” include articles whose authors’ affiliations consisted of both Ecuadorian and foreign institutions.
- “Studies carried out by individual Ecuadorian institutions” includes papers published by researchers affiliated to a single research Ecuadorian institution.

4. The associated scientific or medical field

For a better understanding of the current state of human genetics and human genomics research in Ecuador, all articles were grouped according to their relationship to other scientific fields, medical areas, or human diseases. Our survey considered the following categories which were based on some topics covered during conferences and scientific congresses associated to human and medical genetics [42, 43]:

- Cancer and genetics of solid tumors.
- Genetics of ophthalmologic diseases.
- Genetic diseases, congenital, and rare disorders.
- Genetic engineering and bioinformatic tools.
- Genetics of other complex or multifactorial disorders.
- Genotoxicology.
- Health genetics and genomics, and genetic services.
- Immunogenetics and immunogenomics.
- Metabolic and cardiovascular diseases.
- Neurodevelopmental disorders.
- Human genetic variations associated with parasitic, fungal, bacterial, and viral infections
- Pharmacogenetics and pharmacogenomics.
- Population studies, human phylogeography, and forensic sciences.
- Prenatal diagnosis, genetic counseling, disorders of pregnancy, and menopause.

## Data Availability

The Excel spreadsheet which contains all data about articles retrieved from Scopus, Google Scholar, PubMed, and Web of Science are available upon email request at marlonz1996@hotmail.com (Marlon Zambrano). The remaining data produced or analyzed during the research is available throughout this article.

## Abbreviations

SEGH: Sociedad Ecuatoriana de Genética Humana
DOI: Digital object identifier
WHO: World Health Organization
ReGG: Red de Genética y Genómica
